# Mechanisms for the Direct Electron Transfer of Cytochrome c Induced by Multi-Walled Carbon Nanotubes

**DOI:** 10.3390/s120810450

**Published:** 2012-08-02

**Authors:** Hua-Zhang Zhao, Qian Du, Zhen-Shan Li, Qin-Zheng Yang

**Affiliations:** 1 Department of Environmental Engineering, Peking University, Beijing 100871, China; E-Mails: zhaohuazhang@pku.edu.cn (H.-Z.Z.); lizhenshan@iee.pku.edu.cn (Z.-S.L.); 2 The Key Laboratory of Water and Sediment Sciences, Ministry of Education, Beijing 100871, China; 3 School of Food and Bioengineering, Shandong Institute of Light Industry, Jinan 250353, China; E-Mail: duqian0330@163.com

**Keywords:** direct electron transfer (DET), cytochrome c (Cyt c), multi-walled carbon nanotube (MWCNT), secondary structure

## Abstract

Multi-walled carbon nanotube (MWCNT)-modified electrodes can promote the direct electron transfer (DET) of cytochrome c (Cyt c). There are several possible mechanisms that explain the DET of Cyt c. In this study, several experimental methods, including Fourier transform infrared spectroscopy, circular dichroism, ultraviolet-visible absorption spectroscopy, and electron paramagnetic resonance spectroscopy were utilized to investigate the conformational changes of Cyt c induced by MWCNTs. The DET mechanism was demonstrated at various nano-levels: secondary structure, spatial orientation, and spin state. In the presence of MWCNTs, the secondary structure of Cyt c changes, which exposes the active site, then, the orientation of the heme is optimized, revolving the exposed active center to the optimum spatial orientation for DET; and finally, a transition of spin states is induced, providing relatively high energy and a more open microenvironment for electron transfer. These changes at different nano-levels are closely connected and form a complex process that promotes the electron transfer of Cyt c.

## 1. Introduction

Cytochrome c (Cyt c) is a water-soluble heme protein with a prosthetic iron porphyrin group. It is one of the major members of the biological respiratory chain and acts as an electron carrier, receiving electrons from Cyt c reductase and delivering them to Cyt c oxidase. Cyt c is usually used to modify electrodes to promote direct electron transfer (DET) for biosensors and biofuel cells [1–3]. However, the redox center of Cyt c is deeply embedded in the protein, and the protein is easily denatured upon adsorption onto a bare electrode surface, resulting in extremely slow electron-transfer kinetics and a poor response. To overcome these problems and promote fast electron transfer, electron mediators, such as Santa Barbara Amorphous (SBA-15), gold nanostructures, and carbon nanotubes (CNTs), have been used with Cyt c to modify substrate electrodes [4–8]. DET between Cyt c and electrodes has been observed with the help of the electron mediators.

The DET of Cyt c is a very complex process that has long been disputed. It is thought that the use of electron mediators overcomes some of the obstacles of DET. Electron mediators can provide a suitable surface at the electrode-solution interface, which can preserve the bioactivity of Cyt c and reduce the interfacial resistance between Cyt c and the electrode surface [4–11]. Partially unfolded structures of Cyt c and a shorter tunneling distance between Cyt c and the electrode are also induced by electron mediators [9].

Cyt c probably undergoes conformational changes during the electron transfer process [12]. Investigating these changes will provide insight into the DET mechanism. The secondary structure content changes when Cyt c is absorbed on a single-wall carbon nanotube—modified glassy carbon (GC) electrode [13]. These changes in secondary structure lead to heme exposure, which may influence DET [14,15]. The orientation and symmetry changes of the heme prophyrin ring on the electrode surface induced by electron mediators (such as SBA-15, gold nanostructures, and nitrogenous bases) also play an important role in DET [4,8,16]. In addition, changes in heme spin states affect the electronic activity of heme, which is usually closely related to electron transfer [17–20].

The above-mentioned studies used different electron mediators and focused on a single level of Cyt c (secondary structure, spatial orientation or spin state). It is not clear, however, whether the above-mentioned conformational changes coexist at different levels and how they relate to one another for a single electron mediator.

In recent decades, increasing attention has focused on CNTs due to their unique electronic properties and extremely high superficial volume ratio, which is useful for electron-transfer reactions. CNT-modified electrodes have been used in electrocatalytic reactions [21–23], and many studies have shown that DET can occur between CNTs and various biomolecules including Cyt c [5,7,24–26].

This paper aims to investigate the DET mechanism of Cyt c induced by electron mediators (for example, multi-walled carbon nanotubes, MWCNTs) at different levels. Cyclic voltammetry (CV), Fourier transform infrared (FTIR) spectroscopy, circular dichroism (CD), ultraviolet-visible (UV-vis) absorption spectroscopy, and electron paramagnetic resonance spectroscopy (EPR) were used to investigate DET and analyze the conformational changes of Cyt c. The DET mechanisms of Cyt c induced by MWCNTs were analyzed at different levels.

## 2. Experimental Section

### 2.1. Chemicals

Horse heart Cyt c (MW 12384, Sigma, St. Louis, MO, USA) was used without further purification. MWCNTs were purchased from Shenzhen Nanotech Port (Shenzhen, China). Prior to use, the MWCNTs were pretreated by sonication in a mixture of concentrated sulfuric acid–nitric acid (3:1, v/v) for about 4 h, neutralized and filtered with a Minipore membrane (pore size of 0.22 μm in diameter), and dried at 60 °C overnight to obtain purified MWCNTs. Phosphate buffer solution (PBS, 0.1 M, pH 7.0) was prepared from Na_2_HPO_4_ and NaH_2_PO_4_ and always employed as a supporting electrolyte. All other chemicals were of analytical grade. All the solutions were prepared with doubly distilled water.

### 2.2. Preparation of Cyt c/MWCNT Composites

The preparation process of Cyt c/MWCNT composites is summarized as follows: 1 mg of purified MWCNTs was dispersed in 1 mL PBS (0.1 M, pH 7.0) with the aid of 10 min of ultrasonication to give 1 mg/mL black suspension in which 10 mg of Cyt c was added. The mixture was stored at 4 °C for 12 h followed by centrifugation at 18,000 r/min for 10 min and the removal of the supernatant to obtain Cyt c/MWCNT nanocomposites. The Cyt c/MWCNT composites were washed with distilled water to remove the loosely adsorbed Cyt c molecules and were dried under a frozen vacuum. The Cyt c/MWCNT composites were characterized by FTIR, CD, UV-vis and EPR spectroscopy.

### 2.3. Preparation of Cyt c/MWCNT Modified GC Electrode

The GC electrode (3 mm in diameter) was polished sequentially with slurries of 0.3 and 0.05 μm alumina to mirror and washed with double-distilled water and ethanol in an ultrasonic bath for 1 min. After treatment with acid, MWCNTs were dispersed with the aid of ultrasonic agitation in 10 mL of DMF to give a 1 mg/mL black suspension. The GC electrode was coated by casting 15 μL of suspension of the MWCNTs and dried under an infrared lamp. The above steps were repeated, and an MWCNT-modified electrode was obtained.

Next, Cyt c (10 mg) was dissolved in PBS (1 mL, 0.1 M). The Cyt c solution (20 μL) was sprayed and deposited onto the MWCNT-modified electrode surfaces and dried at room temperature for 4 h to obtain a Cyt c/MWCNT modified electrode. A Cyt c/MWCNT modified electrode was obtained and stored at room temperature after 5 μL of 0.5% Nafion solution was cast onto the modified electrode surfaces.

Prior to use, the Cyt c/MWCNT modified GC electrode and the bare GC electrode were activated using consecutive cyclic potential scans performed in 0.1 M PBS (pH 6.24) for 1.5 min (potential range from 1.5 to −1.0 V, scan rate of 1 V/s).

### 2.4. Characterization and Electrochemical Measurements

Fourier transform infrared (FTIR) spectra made in ATR mode were recorded using a NICOLET iN10 MX FT-IR spectrophotometer (Thermo Scientific, West Palm Beach, FL, USA) to characterize the functionalized MWCNTs and Cyt c/MWCNT nanocomposites in dry state. For each sample, a total of 128 scans at a resolution of 4 cm^−1^ were collected. To investigate the changes in secondary structures of Cyt c immobilized on MWCNTs, the Gaussian curve-fitting method conducted by PeakFit 7.2 software was used to deconvolute the FTIR spectra and Origin 6.0 software was used to calculate the ratios of various secondary structure elements according to their integrated areas.

CD spectra were recorded from 200 to 240 nm with a 0.5 s response and 20 nm/min scanning speed on a JASCO J-715 (Tokyo, Japan) spectropolarimeter using solution with protein concentrations of about 0.3 mg/mL for far-UV regions. The spectra were collected and averaged over 3 scans, using quartz cells of 1.0 mm optical path length. The results are expressed as molar ellipticity, [θ] (deg·cm^2^·dmol^−1^). According to the Yang-Chen formula [27], the ratios of the second ary structure elements (a-helix, b-sheet, turns, and random coils) were calculated using the software package J-715 for Windows Secondary Structure Estimation (Version 1.0).

UV-vis absorption spectra were collected on a BWS003 UV-vis transmittance/reflectance spectrophotometer (B&W Tek Inc., Newark, DE, USA). The UV-vis absorption spectrum of the solution was recorded by an Agilent 8453 UV-vis near-infrared spectrophotometer (Agilent Instruments, Englewood, CO, USA).

EPR spectra were recorded on a Bruker EMX spectrometer (Rheinstetten, Germany). The conditions for EPR measurements were as follows: frequency, 9.6 GHz; power, 3 mW; modulation amplitude, 10 G; modulation frequency, 100 kHz; and temperature, 293 K. As the short time stability of the magnetic field is 5 mG, all the *g* factors have a maximum standard deviation of ±0.17. The high-spin signal at *g* = 6 was quantified by double integration with the lower integration limit taken below the low-fiby end of the spectrum and the upper limit at a field corresponding to a g value of 4.67.

## 3. Results and Discussion

### 3.1. Direct Electrochemistry of Cyt c on the MWCNT-Modified GC Electrode

**Figure 1.**
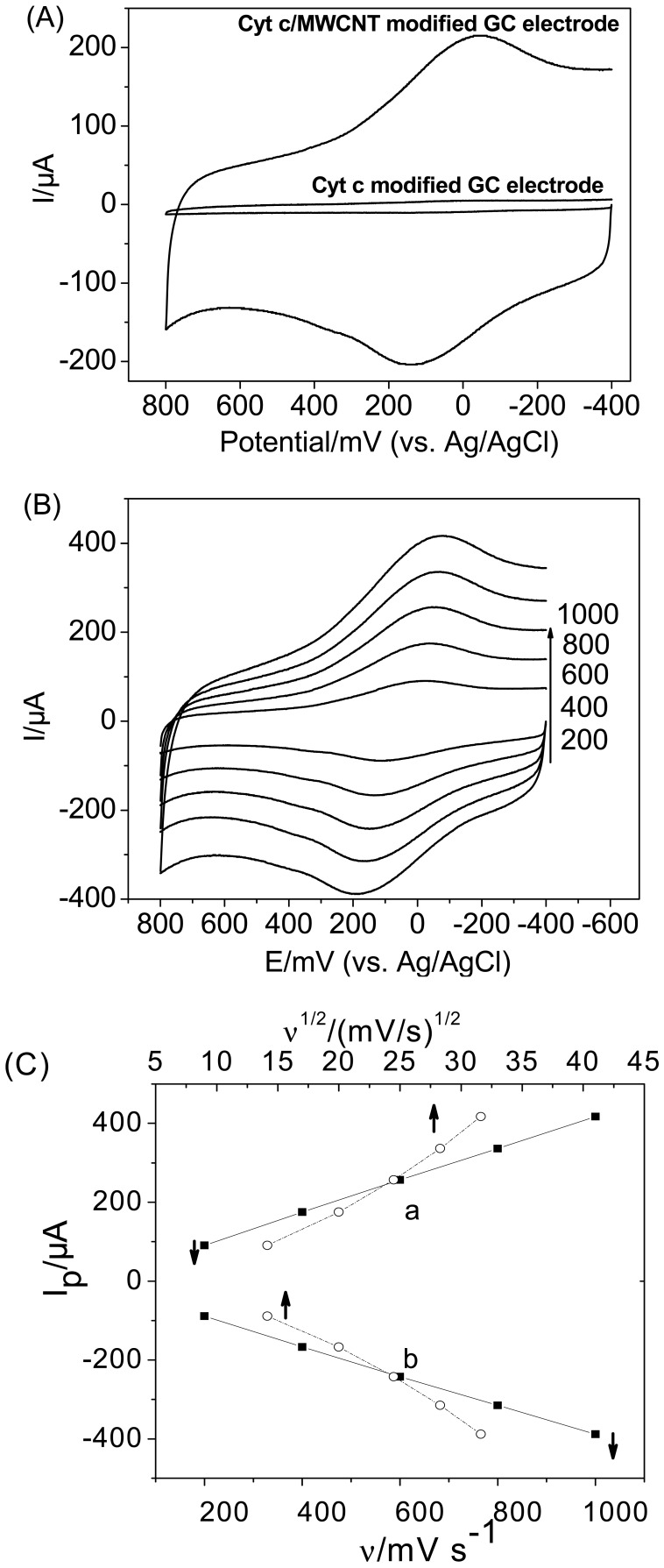
Cyclic voltammograms corresponding to (**A**) Cyt c with and without MWCNTs (scan rate: 50 mV/s); (**B**) Cyt c on the MWCNT-modified GC electrode at various scan rates: 200, 400, 600, 800, and 1,000 mV/s. (**C**) The dependence of (a) the anodic peak currents and (b) the cathodic peak currents on the scan rates and square root of scan rates. The activation potential window of the electrode is 1.5 V–1.0 V. The supporting electrolyte was 1 M PBS (pH 6.07) containing 0.1 M NaCl.

CV experiments were conducted to clarify the DET between Cyt c and the MWCNT-modified electrode in PBS. As shown in Figure 1(A), a pair of well-defined redox peaks appeared at the MWCNT-modified GC electrode with the formal potential of 46 mV, which is almost in agreement with the reported formal potential of Cyt c on SWCNTs [13]. However, no peaks were apparent at the bare GC electrode. This suggests that the thin layer of MWCNT on the GC electrode assists in the DET from the active site of Cyt c to the electrode and that, in the absence of the MWCNTs, no electron transfer occurs at the electrode. This conclusion is consistent with other results of CNT-modified electrodes [28–33].

Figure 1(B,C) illustrates the CVs of Cyt c on the MWCNT-modified GC electrode at various scan rates. The anodic and cathodic peak currents changed linearly with the scan rate in the range of 200 to 1,000 mV/s (The correlation coefficients for the anodicand cathodic peak currents are both greater than 0.9997), while I_p_ *vs.* ν^1/2^ curves show an upward inclination, indicating that the electrode process is controlled by a surface-bound species [34,35]. Furthermore, the peak separations ranging from 135 mV at a scan rate of 200 mV/s to 267 mV at 1,000 mV/s indicate that the adsorbed Cyt c displayed a quasi-reversible electron-transfer process on MWCNTs.

### 3.2. FTIR Spectroscopy

**Figure 2.**
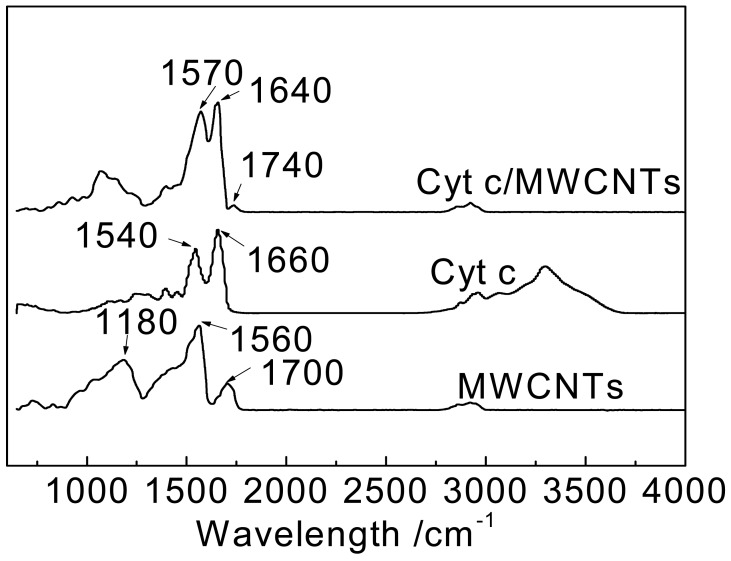
FTIR spectra of MWCNTs, Cyt c, and Cyt c/MWCNTs.

FT-IR spectroscopy was utilized to investigate the environmental and structural changes of Cyt c immobilized on MWCNTs to understand the molecular reaction mechanism. The FTIR spectra of MWCNTs, Cyt c, and Cyt c/MWCNTs are shown in Figure 2.

For the MWCNTs after acidification, the peaks at 1,700 and 1,560 cm^−1^ in the IR spectrum of MWCNTs suggest the presence of carboxyl and carboxylate groups, respectively, while the peak at 1,180 cm^−1^ corresponds to the C–O bond. The FTIR spectrum of Cyt c shows two characteristic adsorption peaks at 1,660 cm^−1^ and 1,540 cm^−1^, corresponding to the amide I and amide II adsorption bands of protein molecules, respectively [4,36]. The amide I vibration is caused by the C=O stretching vibration of peptide linkages in the protein's backbone, and the amide II vibration results from a combination of N-H in-plane bending and C-N stretching vibrations of the peptide groups. They are widely used to monitor changes in secondary structure. The FTIR spectrum of Cyt c immobilized on MWCNTs also shows amide I and amide II adsorption bands, but they shift to some extent. The amide I maximum shifts to lower frequencies from 1,660 to 1,640 cm^−1^, and the amide II maximum shifts to higher frequencies from 1,540 to 1,570 cm^−1^, while the small shift values also indicate that Cyt c retains its natural biological activity.

**Figure 3.**
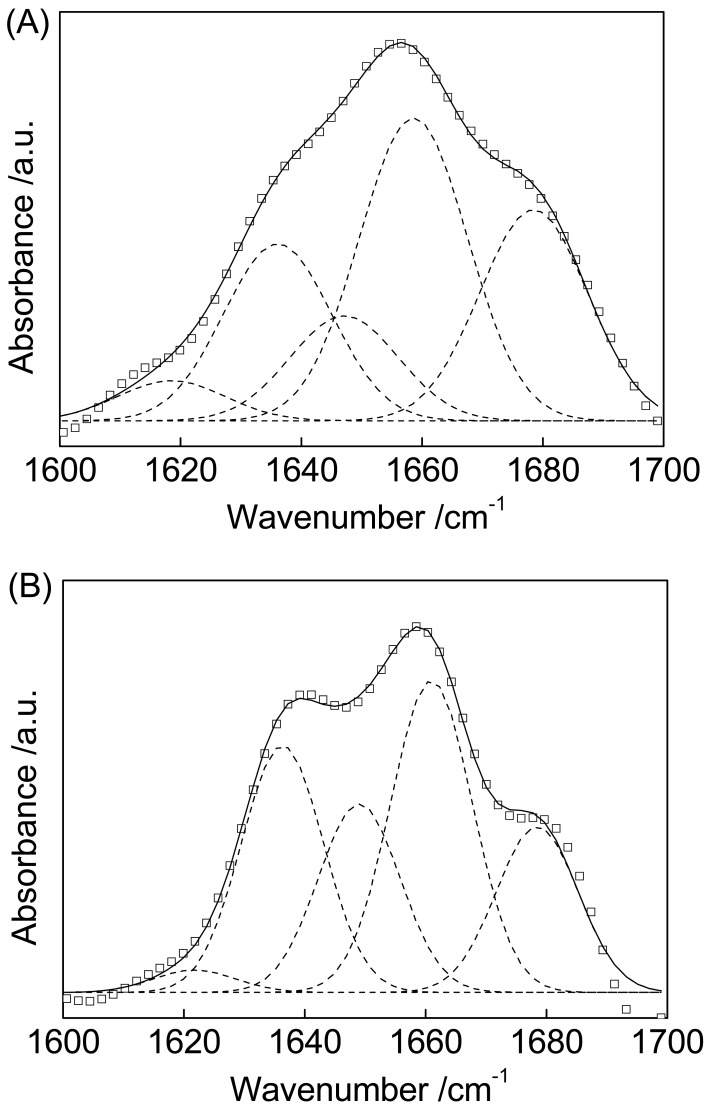
Amide I band peak fitting in the original FTIR spectra for Cyt c (**A**) and Cyt c/MWCNTs (**B**). The original envelope (□), the component bands (dashed line), and the generated envelope (solid line) are shown, respectively.

The slight shift of the amide I and amide II bands of Cyt c absorbed on MWCNTs indicates a corresponding change in secondary structure. Deconvolution of the amide I band of Cyt c before and after absorption onto MWCNTs followed by curve fitting with a Gaussian function yields several distinct underlying absorption bands shown in Figure 3.

Figure 3(A) depicts the spectral decomposition of amide I for Cyt c. The bands at 1,618 was assigned to side chain interactions not associated with the secondary structure [37]. The 1,639 cm^−1^ peak was assigned to beta-sheet structures, which made up 21% of the total area of the amide I band of Cyt c. Alpha-helical structures are characterized by a band at around 1,659 cm^−1^; this component accounts for 36% of the total area of the amide I band. Bands at 1,647 cm^−1^ and 1,678 cm^−1^ correspond to random structures and turns, respectively. These structures make up 13% and 25% of the protein, respectively.

Figure 3(B) illustrates the spectral decomposition of amide I for Cyt c immobilized on MWCNTs. Compared with Figure 3(A), the alpha-helix peak position shifts from 1,659 cm^−1^ to 1,661 cm^−1^ while the peaks for the beta-sheet structures almost unchanged. The alpha-helical content decreases from 36% to 33% while the beta-sheet content increases from 21% to 26%. These data suggest that the interaction between Cyt c and MWCNTs results in the conversion of alpha-helices to beta-sheets.

### 3.3. CD Spectroscopy

The FTIR spectra focus on the changes in functional groups, and they provide preliminary conformational analyses of solid-state proteins. CD is an advanced tool to investigate the conformational changes of proteins in a dilute aqueous solution. In the present work, CD was applied to give an overview of the secondary structures of Cyt c interacting with MWCNTs.

**Figure 4.**
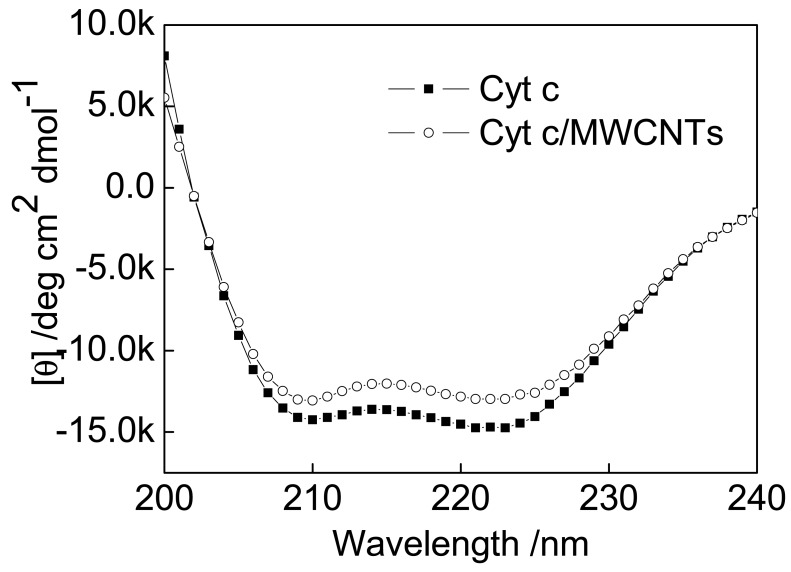
Far-UV CD spectra of Cyt c and Cyt c/MWCNTs.

The far-UV CD spectra of native Cyt c and Cyt c immobilized on MWCNTs exhibit two negative peaks at 208 and 221 nm resulting from the chirality of alpha-helical structures (Figure 4). These results are similar to those of previous reports [38,39]. Absorption in the region below 240 nm is due primarily to the peptide bond. Therefore, it can be concluded from the CD spectra that the structure and activity of Cyt c after being treated with MWCNTs are essentially the same as in the native protein. The secondary structure content of the native and the treated Cyt c were calculated and the results are compared with the corresponding results of FTIR spectra as follows.

Both FTIR and CD spectra reveal a decrease in alpha-helices (FTIR: from 36% to 33%; CD: from 26% to 23%) and an increase in beta-sheets (FTIR: from 21% to 26%; CD: from 12% to 13%). These results further confirmed that the presence of MWCNTs cause a decrease in alpha-helices in Cyt c.

The alpha-helices are mainly located on the exterior of the protein and are more compact than other secondary structures. As a result, the alpha-helical structure is one of the barriers for electron transfer [33]. Reducing the alpha-helical content loosens the protein shell and exposes the active site. Thus, it is easier to approach the active center, facilitating DET.

### 3.4. UV-Vis Absorption Spectroscopy

**Figure 5.**
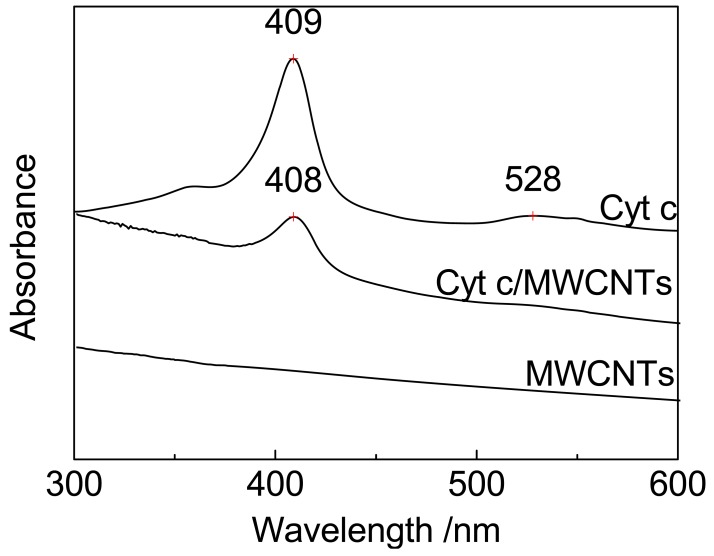
UV-vis absorption spectra of MWCNTs, Cyt c, and Cyt c/MWCNTs.

The spatial orientation of the heme porphyrin ring, which may be a factor that affects electron transfer, was investigated by UV-vis absorption spectroscopy, as shown in Figure 5. There is no absorption observed in Figure 5 for the black MWCNT particles. The spectrum of Cyt c exhibits two obvious UV absorption peaks at 409 and 528 nm, corresponding to the Soret band and Q-band of Cyt c, respectively. The spectrum of Cyt c immobilized on MWCNTs also shows a Soret band with a decrease in intensity and a hypsochromic shift of only 1 nm to 408 nm.

The Soret band results from the additive effects of the transition dipole moments of the two orbital excitations a_1u_-e_g_ and a_2u_-e_g_ of π-π* transitions of the porphyrin ring in Cyt c; therefore, the intensity of the Soret band reflects the symmetry of the porphyrin ring (the spatial orientation) [4,13,39,40], and the shift of the Soret band reflects the conformational changes of the heme microenvironment [4,41].

For Cyt c immobilized on MWCNTs, the small shift of the Soret band suggests that the interaction between Cyt c and MWCNTs has not destroyed the biological activity of Cyt c, but has changed its microstructure. These results agree with previous studies on Cyt c absorbed on SBA-15 and gold nanostructures [4,8,42]. It is suspected that some intermediate states of the heme porphyrin ring are produced during the electron transfer process, and the π-π* transition is decreased [16]. As a result, the intensity of the Soret band decreases as shown in Figure 5. As the dipole direction is consistent with the spatial orientation [43], these intermediate states may correspond to some transient spatial orientations of the heme porphyrin ring, which are characterized by the changes in the symmetry of the porphyrin ring. The transient spatial orientations probably facilitate the electron transfer of Cyt c [4,44]. As suggested above, it is possible that the MWCNT surface provides a more favorable spatial orientation of the heme porphyrin ring for DET.

From another point of view, the shift and the intensity decrease of the Soret band may result from the decrease in the hydrophobic nature of Cyt c. The hydrophobic nature is closely related to the amount of alpha-helices due to their compactness and specific location in the protein exterior [4,15]. The FTIR and CD results indicate that the alpha-helical content decreases when Cyt c interacts with MWCNTs, which results in a decrease in the hydrophobic nature of Cyt c. The protein orientation may also be optimized by a decrease in hydrophobic interactions [4], and DET would be enhanced with the appropriate orientation.

### 3.5. EPR Spectroscopy

**Figure 6.**
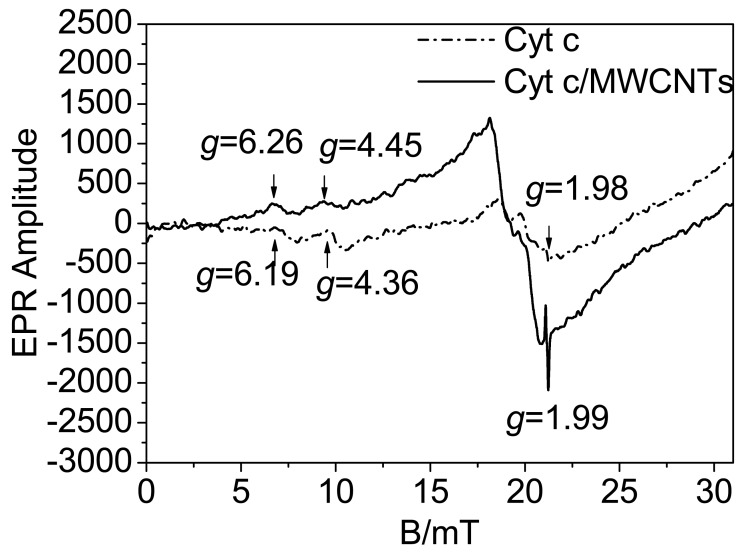
EPR spectra of Cyt c and Cyt c/MWCNTs.

As has been reported, the spin state equilibrium of the heme iron could modulate both substrate binding and the oxidation-reduction reactions of the cytochrome [19], and electron transfer is always accompanied by a change in iron spin state [20]. We speculate that the spin state influences the electron transfer activity. EPR spectra were utilized to investigate the conversion of the spin states, and the heme iron axial ligation is shown in Figure 6. As shown in Figure 6, the signals of the native Cyt c represent a mixture of high and low spin states, characterized at g = 6.19 and g = 4.36, respectively [17]. The signal at g = 1.98 is from the strong Fe-S ligation in the heme redox center. Fe-S ligation is important for the bioactivity of Cyt c, and damage to the Fe-S ligation will cause deactivation of the protein. When Cyt c interacts with MWCNTs, the Fe-S ligation signal nearly stay the same, suggesting that Fe-S ligation has not been destroyed, and the protein retains its bioactivity in this process. The low spin signal has a red shift to g = 4.45 along with a decrease in intensity while the high spin signal is observed at g = 6.26 along with an increase in intensity. This result suggests that the interaction between Cyt c and MWCNTs induces a transition of the heme iron from a low spin state to a high spin state. Cyt c has six heme iron coordination sites [45]. Low spin hemes are six-coordinated, which means that the six coordination sites of the heme iron are occupied by intrinsic ligands. In contrast, high spin heme compounds are formally five-coordinated, leaving a coordination site open for the binding of extrinsic ligands [19]. X-ray structures of model compounds and proteins have shown that the high-spin iron atom sits about 0.5 Å out of the porphyrin plane while the low-spin iron is in-plane [46,47]. Accordingly, high spin states have a relatively high energy and provide a more open microenvironment for electron transfer than low spin states. According to the observed transition to high spin states as shown in Figure 6, the active center of the heme ring is in a state that facilitates DET. In addition, the transition of the spin states is accompanied by significant nuclear reorganization [20] to optimize the orientation of the heme ring for DET, which is similar to the results from the UV-vis spectroscopy.

The above assays confirm that Cyt c goes through the above conformational changes at three levels: secondary structure, spatial orientation, and spin states. The changes in the three levels are not isolated, but closely interrelated; changes in secondary structure provide an exposed active center; changes in the spatial orientation of the porphyrin ring position the active center in a more favorable orientation to receive and transmit electrons; changes in spin states make the active center more appropriate for high electronic activity. As a result, a microenvironment with an open enzyme active center, the correct orientation of the active center, and an appropriate state for the electrons is created by which DET between Cyt c and MWCNT-modified electrodes is achieved and enhanced.

## 4. Conclusions

DET between Cyt c and MWCNT-modified electrodes was achieved in this work. The conformational changes of Cyt c at different nano-levels were investigated using different analytical methods (FITR, CD, UV, and EPR) to study the DET mechanism. First, MWCNTs affect the ratio of secondary structure elements, leading to exposure of the active site. Then, the exposed active center is oriented in the correct orientation for DET. Finally, a transition of spin states is induced to provide relatively high energy and a more open microenvironment for electron transfer. The DET mechanism could apply to other electron mediators and proteins. The understanding of the mechanism would contribute to the development of modified electrodes and biosensors.
